# Flexible memristive devices based on polyimide:mica nanosheet nanocomposites with an embedded PEDOT:PSS layer

**DOI:** 10.1038/s41598-018-30771-5

**Published:** 2018-08-16

**Authors:** Myoung Kyun Choi, Woo Kyum Kim, Sihyun Sung, Chaoxing Wu, Hyoun Woo Kim, Tae Whan Kim

**Affiliations:** 1 Department of Electronics and Computer Engineering, Hanyang University, Seoul, 133-791 Republic of Korea; 2 Division of Materials Science and Engineering, Hanyang University, Seoul, 133-791 Republic of Korea

## Abstract

Flexible memristive devices with a structure of Al/polyimide:mica/poly(3,4-ethylenedioxythiophene) polystyrene sulfonate/indium-tin-oxide/polyethylene glycol naphthalate showed electrical bistability characteristics. The maximum current margin of the devices with mica nanosheets was much larger than that of the devices without mica nanosheets. For these devices, the current vs. time curves showed nonvolatile characteristics with a retention time of more than 1 × 10^4^ s, and the current vs. number-of-cycles curves demonstrated an endurance for high resistance state/low resistance state switchings of 1 × 10^2^ cycles. As to the operation performance, the “reset” voltage was distributed between 2.5 and 3 V, and the “set” voltage was distributed between −0.7 and −0.5 V, indicative of high uniformity. The electrical characteristics of the devices after full bendings with various radii of curvature were similar to those before bending, which was indicative of devices having ultra-flexibility. The carrier transport and the operation mechanisms of the devices were explained based on the current vs. voltage curves and the energy band diagrams.

## Introduction

Extensive investigations concerning flexible organic electronics, including memories, memristors, synaptic devices and nanogenerators, have been performed to improve their performance and efficiency for promising applications in flexible systems. Memristive devices are subjects of great scientific interest because they are highly integrable and can be fabricated using simple process^1–4^. Memristive devices based on various kinds of nanocomposites have been actively investigated to find ways to improve their electrical characteristics, and memristive devices based on hybrid inorganic/organic nanocomposites containing nanomaterials have currently emerged as excellent candidates for promising applications in flexible/wearable memristive devices due to their having high-mechanical flexibility and relatively low-cost^5–9^. Among the several kinds of nanomaterials, two dimensional (2D) materials with a monolayer thickness, such as graphene, are attractive because they have high conductivity, good flexibility, and high transparency. The use of 2D materials is limited by the narrowing of their bandgaps; that narrowing occurs because, precise control of the bandgaps for nanomaterials is very difficult^10–13^. Among the various kinds of 2D materials, a muscovite-type mica bulk has a large band gap, resulting in its having insulating properties. Worthy of note are the observation that a bulk mica layer can be reduced by using a liquid-phase exfoliation method and that the band gap can be reduced by decreasing the number mica layers. Multilayer mica materials have been employed in electronic and optoelectronic devices due to their unique properties^14,15^. On the other hand, the polymer acting as the insulating matrix plays an important role in memristive devices. Although polyimide (PI) was extensively used in electronics due to its strong thermal and mechanical stability, low toxicity, and excellent electrical properties, in various industrial fields, it has recently been replaced by inorganic glass and metal oxides^16–18^. Given the advantages of PI and mica, investigations concerning memristive devices based on PI:mica nanosheet nanocomposites should be important; nevertheless, such investigations, to the best of our knowledge, have not yet been performed.

This paper reports data, obtained both before and after bending, for the electrical bistabilities, the memory stabilities, and the memory mechanisms of ultra-flexible memristive devices based on PI:mica nanosheet hybrid nanocomposites fabricated on flexible polyethylene glycol naphthalate (PEN) substrates. Because the electrical performances might have been impaired due to the weak adhesive force the occurred between the bottom indium-tin-oxide (ITO) electrode and the PI:mica active layer when the electrode was being repeatedly bent, poly(3, 4-ethylenedioxythiophene):poly(styrene sulfonate) (PEDOT:PSS), acting as an adhesive buffer layer, was inserted between the ITO electrode and the active layer^19–21^. The PI:mica nanocomposite were measured by using scanning electron microscopy (SEM), and the current-voltage (I–V) characteristics were measured to confirm the memristive performances. Endurance and retention measurements were performed to clarify the stabilities of the devices under mechanical bending conditions, and the results were used to investigate the stabilities under different degrees of mechanical deformations. Finally, the carrier transport and the operation mechanisms were explained based on the I–V curves and the energy band diagram.

## Methods

An ITO film was used as the bottom electrode, and an Al thin film was used as the top electrode. After the PEDOT:PSS film had been deposited on a PEN substrate, the PI:mica nanocomposite was spin-coated on a PEDOT:PSS film. The PI used in this experiment was prepared by dissolving p-phenylene biphenyltetracarboximide (BPDA-PDA)-type polyamic acid in N-methy1–2-pyrrolidone (NMP) with a concentration of 1 wt%^16,22^. The exfoliated muscovite-type mica nanosheets used in this experiment were prepared by using ultrasonication, centrifugation, and ultrasonication^14^. The ultrasonic treatment was performed for 4 h to obtain a uniform mica nanosheet suspension. After the ultrasonic treatment of the mica nanosheet, the PI solution and the mica suspension were mixed in a mass ratio of 4:1. Then, another ultrasonic treatment was performed at 27 °C for 4 h to achieve a uniform suspension. ITO/PEN substrates were cleaned ultrasonically in acetone, methanol, and deionized water at 27 °C for 20 min each. The substrates were dried by using N_2_ gas. The PEDOT:PSS solution with a concentration of 1.1 wt% was spin-coated onto the cleaned ITO/PEN substrate at 500 rpm for 5 s, 1000 rpm for 15 s, 2000 rpm for 30 s, 1000 rpm 15 s, and 500 rpm 5 s. Then, the sample was thermally annealed at 100 °C for 20 min. The PI:mica solution was spin-coated onto the PEDOT:PSS/ITO/PEN substrate at 500 rpm for 5 s, 1000 rpm for 15 s, 2000 rpm for 30 s, 1000 rpm 15 s, and 500 rpm 5 s, followed by thermal annealing at 100 °C for 20 min to remove the residual solvent on the PI:mica nanocomposite. Al electrodes (diameters: 1 mm; thicknesses: 200 nm) were deposited on the PI:mica layer by using thermal evaporation at a chamber pressure of 1 × 10^−6^ Torr. SEM (NOVA NanoSEM 450 system), transmission electron microscopy (TEM, JEM-2100F), and atomic force microscopy (AFM, XE-100) were used to characterize the device, the as-prepared mica nanosheets, and the PI:mica nanocomposite. The I–V curves of the memristive devices were measured by using a Keithley 2400 digital source meter.

## Results and Discussion

**Figure 1:**
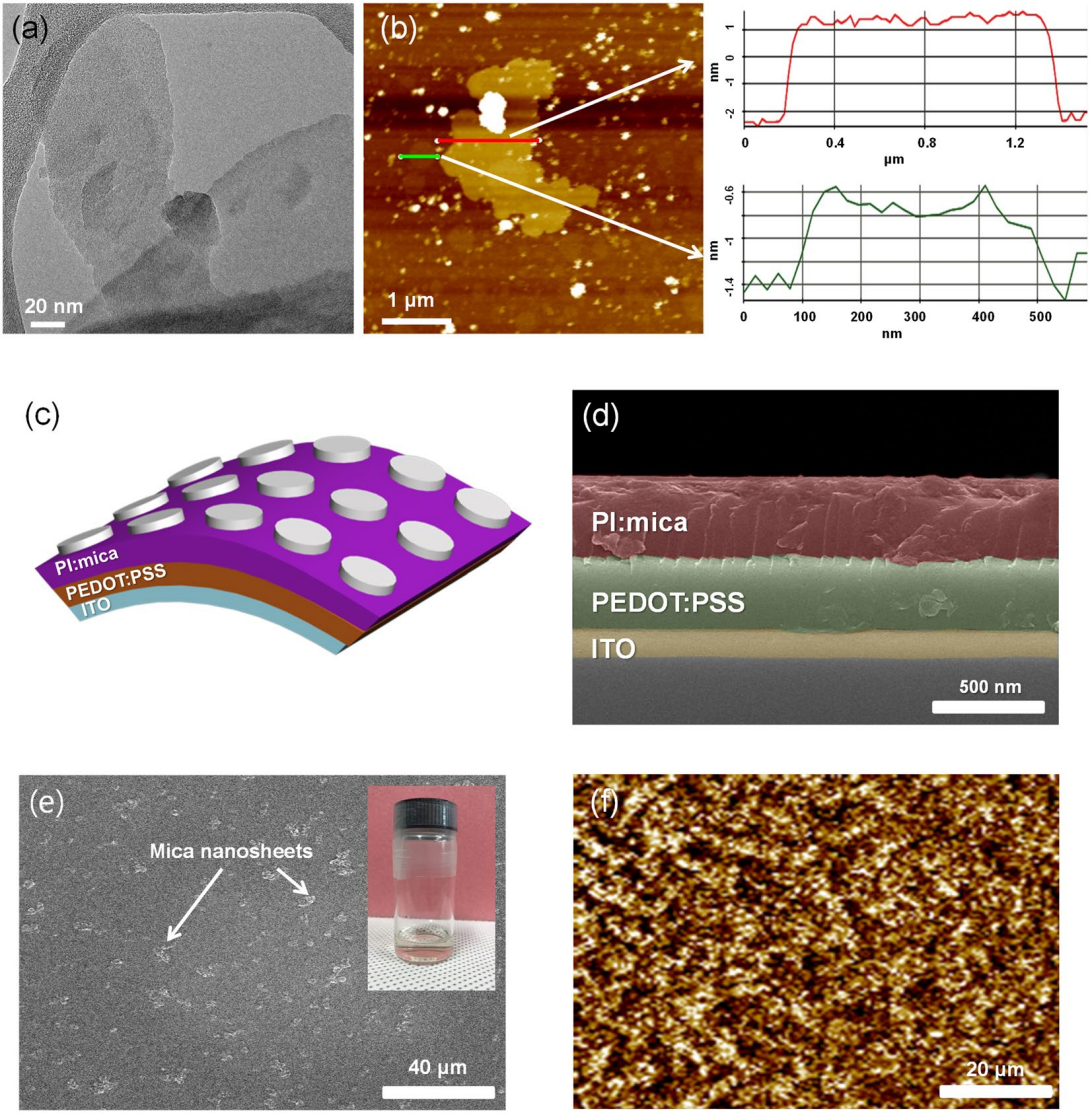
(**a**) TEM image of a mica nanosheet. (**b**) Typical AFM image of mica nanosheets on a Si substrate. (**c**) Schematic diagram of an Al/PI:mica/PEDOT:PSS/ITO device fabricated on a PEN substrate. (**d**) Cross-sectional SEM image of the PI:mica/PEDOT:PSS/ITO device. (**e**) SEM image of the PI:mica surface. Inset is the photo of mica:PI solution. (**f**) Atomic force microscopy image of the PI:mica surface.

Figure 1(a) shows a typical TEM image of the exfoliated mica nanosheets. Characterization by using AFM detected a sheet-like morphology with a thickness ranging from 1 to 4 nm and with a layer number ranging from 1 to 3, as shown in Fig. 1(b)^14^. The thinnest sheets corresponded to monolayer nanosheets. A schematic of the memristive devices is shown in Fig. 1(c,d) shows a cross-sectional SEM image of the memristive device without top electrodes. The SEM image shows that the PI:mica and the PEDOT:PSS layers were precisely deposited on the ITO-coated PEN substrate. The thicknesses of the PEDOT:PSS layers and the PI:mica layer, as determined form the SEM image, were 338 and 424 nm, respectively. Note that after an ultrasonic treatment for 4 h, the exfoliated muscovite-type mica was uniformly mixed with the PI layer in the NMP solvent. As a result, a homogeneous solution of exfoliated mica and PI without any precipitate was obtained, as shown in inset of Fig. 1(e). Because the exfoliated muscovite-type mica was uniformly distributed in the PI solution, the mica nanosheets should be uniformly distributed in the PI:mica layer, as shown in the SEM image in Fig. 1(e). The AFM images in Fig. 1(f) show the roughness of the PI:mica layer. The value of the root-mean-square (RMS) roughness of the PI:mica layer was 5.377 nm.

**Figure 2:**
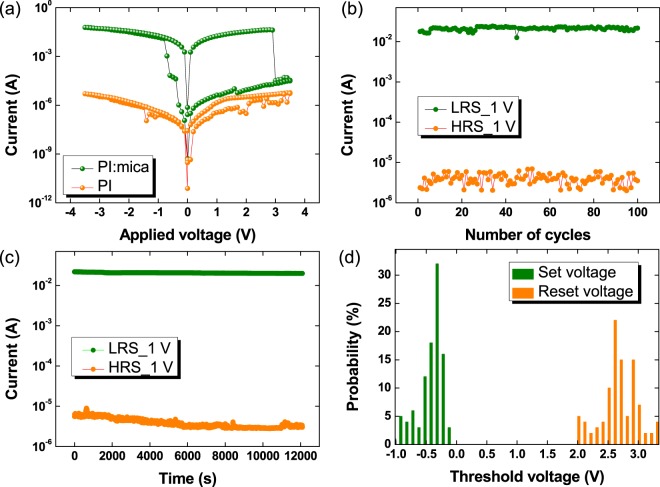
(**a**) Current-voltage curves for the Al/PI:mica/PEDOT:PSS/ITO/PEN devices (black circles) and the Al/PI/PEDOT:PSS/ITO/PEN devices (red circles). (**b**) Endurance characteristics of the Al/PI:mica/PEDOT:PSS/ITO/PEN device before bending at a reading voltage of 1 V. (**c**) Retention characteristics of the Al/PI:mica/PEDOT:PSS/ITO/PEN devices at a reading voltage of 1 V. (**d**) Probability distributions for the ‘Set’ and the ‘Reset’ threshold voltages of the Al/PI:mica/PEDOT:PSS/ITO/PEN devices.

Because the PI:mica nanocomposite forms a uniform structure, the memristive device exhibits stable memory switching characteristics. The I–V curves in Fig. 2(a) show the switching characteristics of the memristive device. The black and the red circles represent the I–V curves of the Al/PI:mica/PEDOT:PSS/ITO device and the Al/PI/PEDOT:PSS/ITO device, respectively. The I–V curve of the Al/PI:mica/PEDOT:PSS/ITO device shows hysteresis characteristics, which is an essential feature of memristive memory devices^23^. Two different resistance states, the high resistance state (HRS) and the low resistance state (LRS), were clearly observed. Initially, the device in its initial state stayed in the HRS. When the negative bias voltage reached −0.3 V, the current increased from 1.03 × 10^−6^ to 1.22 × 10^−2^ A, indicative of the switch from the HRS to the LRS. The HRS-to-LRS transition corresponds to a set process in a memory cell. After the device had switched from the HRS to the LRS, it stayed in the LRS. However, when the positive bias voltage was increased to 3 V or more, the current decreased from 4.15 × 10^−2^ to 3.29 × 10^−5^ A, as shown in Fig. 2(a), indicative of a switch from the LRS to the HRS. The LRS-to-HRS transition corresponds to a reset process in a memory cell. The LRS/HRS current ratio of the device containing mica at a reading voltage of 1 V was approximately 4.28 × 10^3^, as shown by the black circles in Fig. 2(a). However, the device without the mica nanosheets did not clearly show any the memory characteristics, as shown by the red circles in Fig. 2(a). Therefore, one can conclude that the mica nanosheets play an important role in the appearance of memory characteristics for the Al/PI:mica/PEDOT:PSS/ITO device. One should note that mica concentration of 1 wt% used in this work is the concentration required for optimal device performance. When the mica concentration was lower than 1 wt%, the ON/OFF ratio of the device gradually increased with increasing mica concentration. This behavior indicated that the mica was acting as carrier trap sites in the device, resulting in an increase in the number of electrons trapped in the active layer. However, the ON/OFF ratio decreased with increasing mica concentration when the mica concentration was higher than 1 wt%, which might results from the aggregation of mica nanosheets in the active layer.

Figure 2(b) shows the endurance reliability results for the Al/PI:mica/PEDOT:PSS/ITO device. The endurance reliability measurements were carried out at room temperature. The memristive device could be repeatedly switched between the LRS and the HRS for 100 cycles without any noticeable deterioration in its performance. Figure 2(c) shows the retention performance of the device. The retention performances show the variations of the currents in the LRS and in the HRS as functions of time. As can be seen, a stable LRS/HRS current ratio was maintained for a time longer than 1 × 10^4^ s, indicating that the device remained stable even during long operations. Figure 2(d) shows the probability distributions of the reset voltage (V_reset_) and the set voltage (V_set_) for the device. The values of the V_reset_ for the device were dominantly distributed between 2.0 and 3.5 V, and those of the V_set_ were mainly dispersed between −1.0 and −0.1 V. Therefore, the V_set_ and the V_reset_ for the devices could be defined as −1.0 V and 3.5 V, respectively.

**Figure 3:**
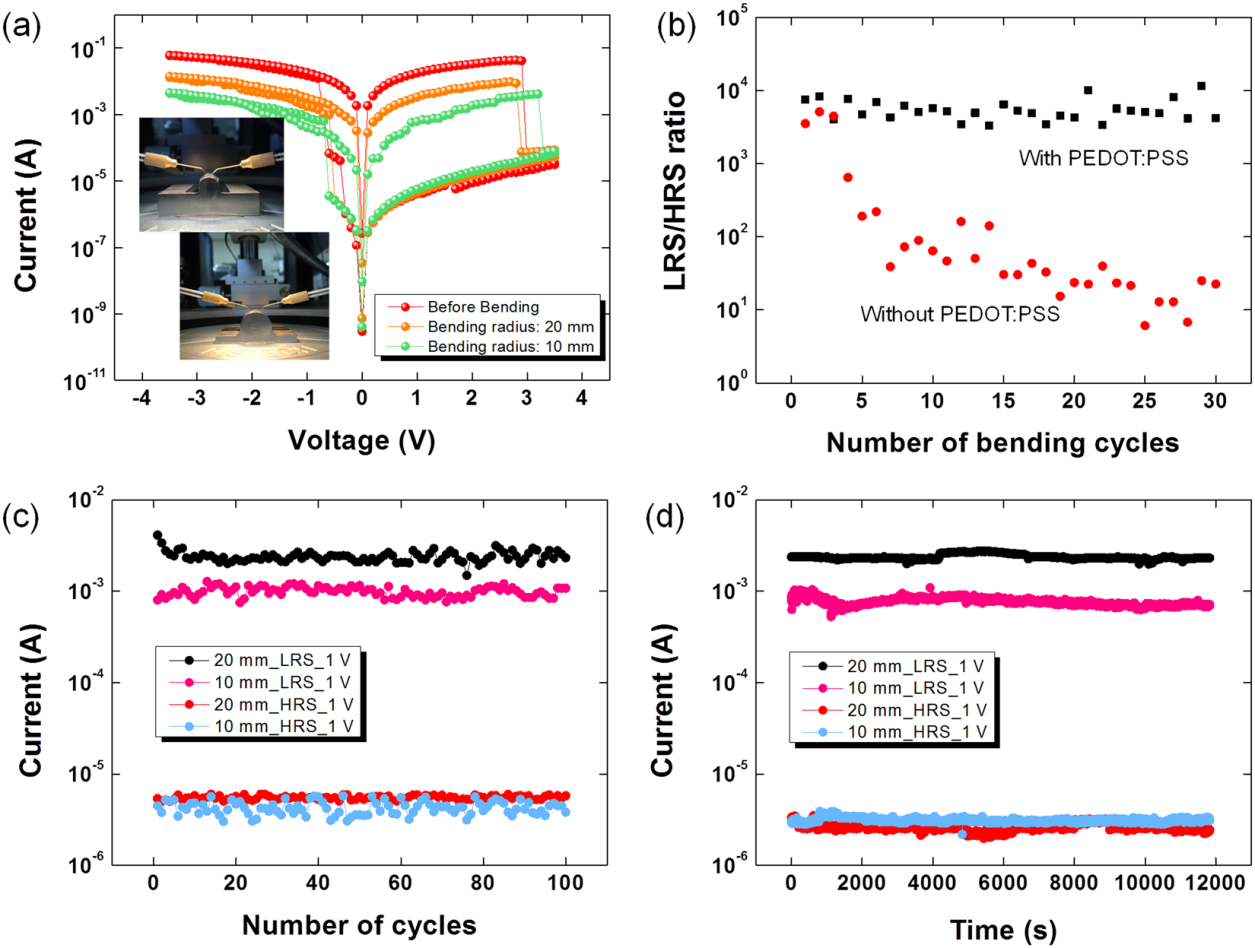
(**a**) Current-voltage curves for the Al/PI:mica/PEDOT:PSS/ITO/PEN devices for bending radii of 0, 10, and 20 mm. (**b**) Endurance characteristics as a function of the number of bending cycles of the device with and without PEDOT:PSS layer, respectively. (**c**) Endurance characteristics after bending of the Al/PI:mica/PEDOT:PSS/ITO/PEN devices at a reading voltage of 1 V. (**d**) Retention characteristics after bending of the Al/PI:mica/PEDOT:PSS/ITO/PEN devices at a reading voltage of 1 V.

The I–V curves for the Al/PI:mica/PEDOT:PSS/ITO devices before and after bending with bending radii of 10 and 20 mm are shown in Fig. 3(a). The I–V results demonstrate that the memory performances of the devices bent at different radii were similar to those of the device in its flat state, indicative of strong bending stability. The LRS/HRS current ratios of the devices after bending at radii of 20 and 10 mm were approximately 7.67 × 10^2^ and 1.08 × 10^2^, as shown by the orange and the green circles in Fig. 3(a), respectively. Because the bending of the device might break the ITO electrode due to its fragility, resulting in a remarkable decrease in the LRS current, the LRS/HRS current ratio for the curving device should be reduced in comparison with that before bending^24^. The PEDOT:PSS film was used to improve the stability of the device when suffered to mechanical bending. The PEDOT:PSS thin film was used to improve the stability of the device while it was being subjected to repeated mechanical bending. As is well known, the poor wettability of polymer layers on ITO electrodes can lead to poor stability of the device because of an unfavorable disparity in the surface energy^25^. Especially, the repetitive mechanical curving could cause the device’s performance to deteriorate. Therefore, a modified layer between the ITO electrode and the active film is important to the improvement of the stability of flexible devices. The LRS/HRS ratio for the device without the PEDOT:PSS layer decreased under repeated bending, as shown in Fig. 3(b). However, the device with the PEDOT:PSS layer could be stably operated under repeated bending without any noticeable degradation. Figure 3(b) shows the endurance reliability of the device after bending. The device could be repetitively operated without any noticeable variation, regardless of bending, indicating that the electrical characteristics of the device were very stable. Figure 3(c) show the retention performances of the device after bending. The device maintains the LRS/HRS ratio for at least 1 × 10^4^ s after bending, indicating that, regardless of bending, the device was very reliable, even for lengthy operations.

**Figure 4:**
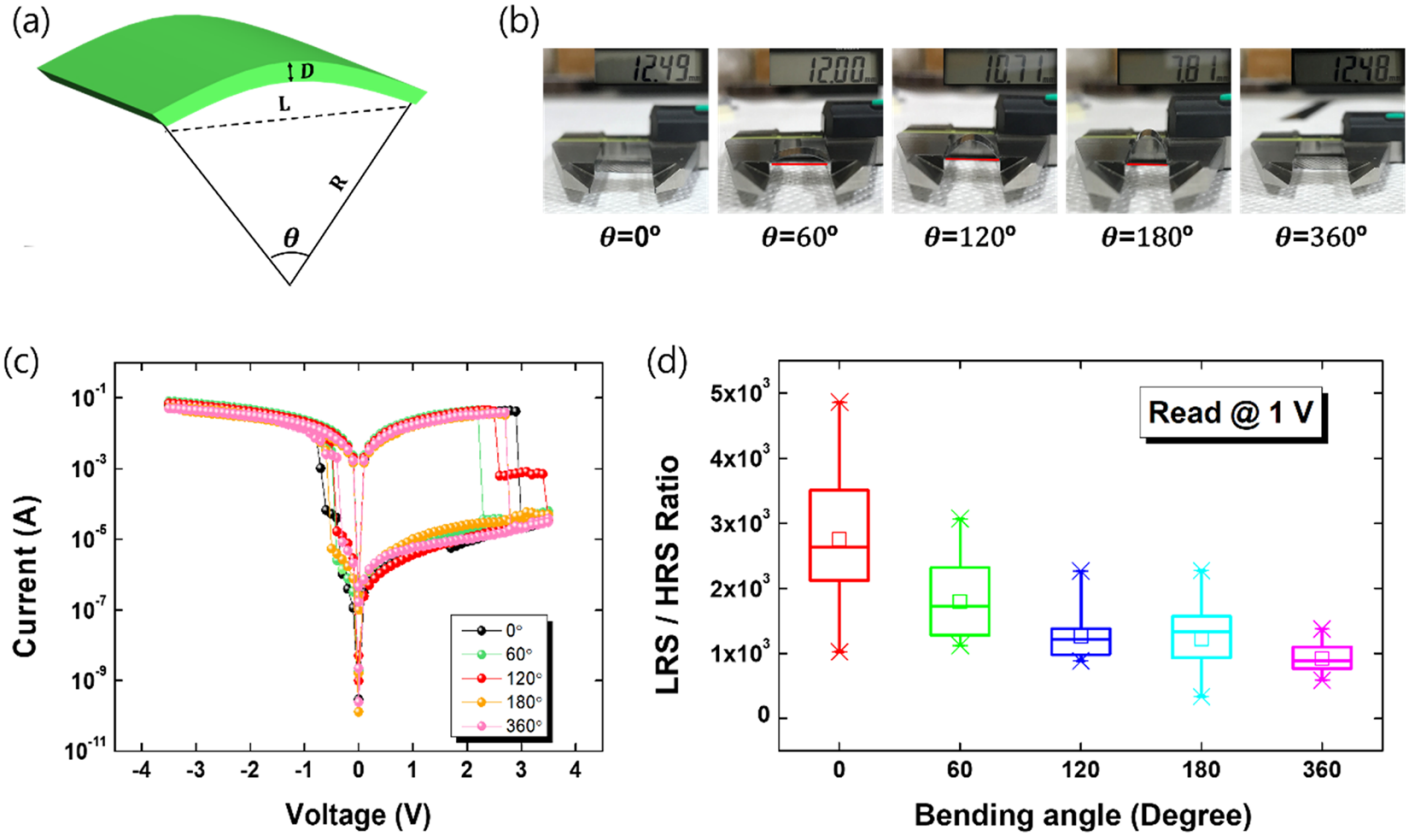
(**a**) Schematic illustration of the flexible ITO-coated PEN substrate, where θ is the corresponding central angle after bending. (**b**) Photographs of the Al/PI:mica/PEDOT:PSS/ITO/PEN devices being bent. (**c**) Current-voltage curves for the devices after bending were compared with those before bending (flat state). (**d**) Bending-angle distribution of the LRS/HRS ratios for the Al/PI:mica/PEDOT:PSS/ITO/PEN devices.

The device was repeatedly bent 30 times at different angles in order to further investigate characteristics of the flexible memristive device, and the I–V characteristics after bending were compared with those before bending (flat state). Figure 4(a) presents the geometry of the device when it is bent, where R is the bending radius, D is the thickness of the device, θ is the central angle, and L is the distance of the line connecting the two endpoints, which is the chord. The bending angle can be determined from the value of L after bending, as shown in Fig. 4(b,c) shows that the electrical properties of the device did not vary significantly as a result of its being bent. Figure 4(d) shows the distribution of the LRS/HRS ratios for 30 devices with bending different bending angles.

**Figure 5:**
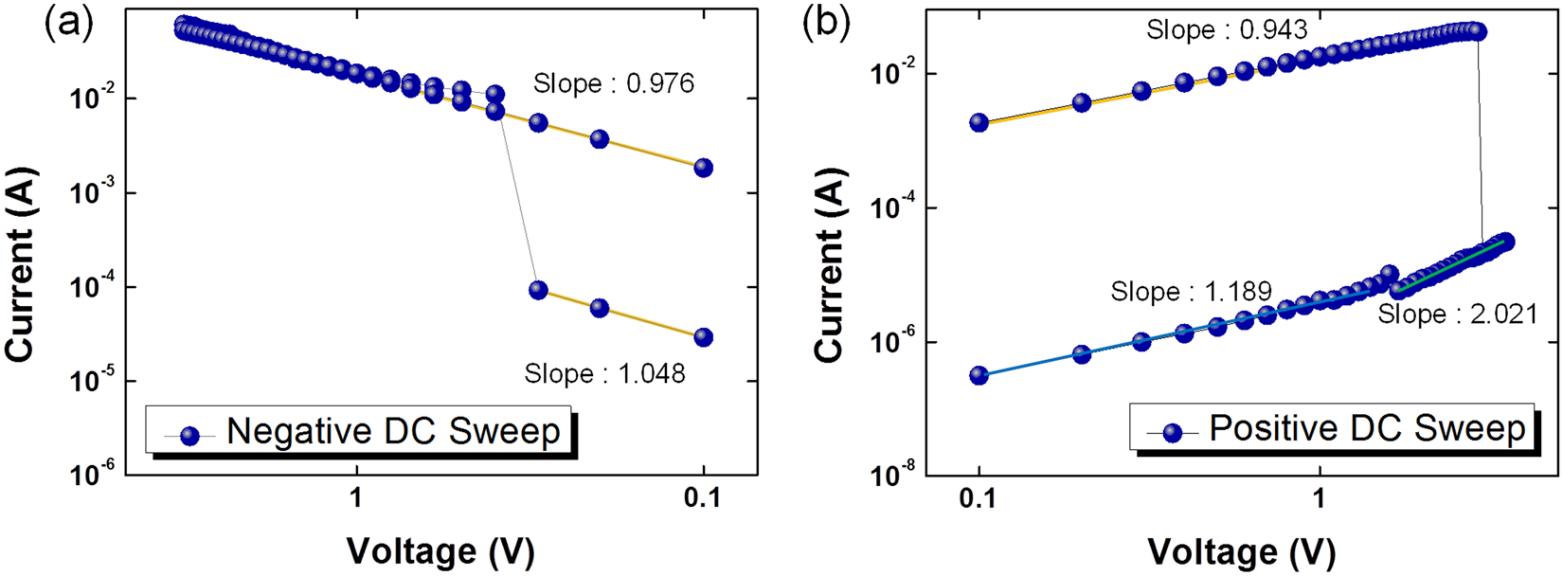
Log-log plots, with fitting lines, of the current as a function of the applied voltage for the Al/PI:mica/PEDOT:PSS/ITO/PEN devices under (**a**) negative DC voltage sweeps and (**b**) positive DC voltage sweeps.

Several conduction models, as determined from fittings of the I–V curves on a log-log scale, can be used to clarify the carrier transport and memory mechanisms of the memristive device^26^, and Fig. 5(a,b) show the results of such fittings. When a set bias voltage was applied to the device, the device switched to the LRS, as shown in Fig. 5(a). The carrier transport mechanism of the device under negative bias voltage exhibited a linear relationship between I and V, indicative of Ohmic conduction. Thus, one can speculate that a conduction filament was formed in the PI:mica layer when the device stayed in the LRS^27^. However, the carrier transport mechanisms in the HRS were found to be related to both Ohmic conduction and space-charge-limited-current (SCLC) conduction. The slope of the fitting curve at low voltage for the HRS was approximately 1, as shown in Fig. 5(b), indicative of Ohmic conduction. However, the charge transport mechanism under low applied bias voltage was controlled by thermally generated free electrons, with the number of electrons injected from the electrode increasing with increasing electric field. Therefore, the carrier transport mechanism is dominantly affected by the injected electrons, resulting in their capture in trap sites. As a result, the current increased rapidly in a manner consistent with Child’s square law (I~V^2^). From the Ohmic region to the Child’s square region, the threshold voltage coincides with a transition from a trap-unfilled state to a trap-filled state^28,29^. Thus, immediately after the transition to the HRS, the charge transport mechanism exhibits a SCLC conduction behavior, with a slope of approximately 2, because some electrons remain trapped.

**Figure 6:**
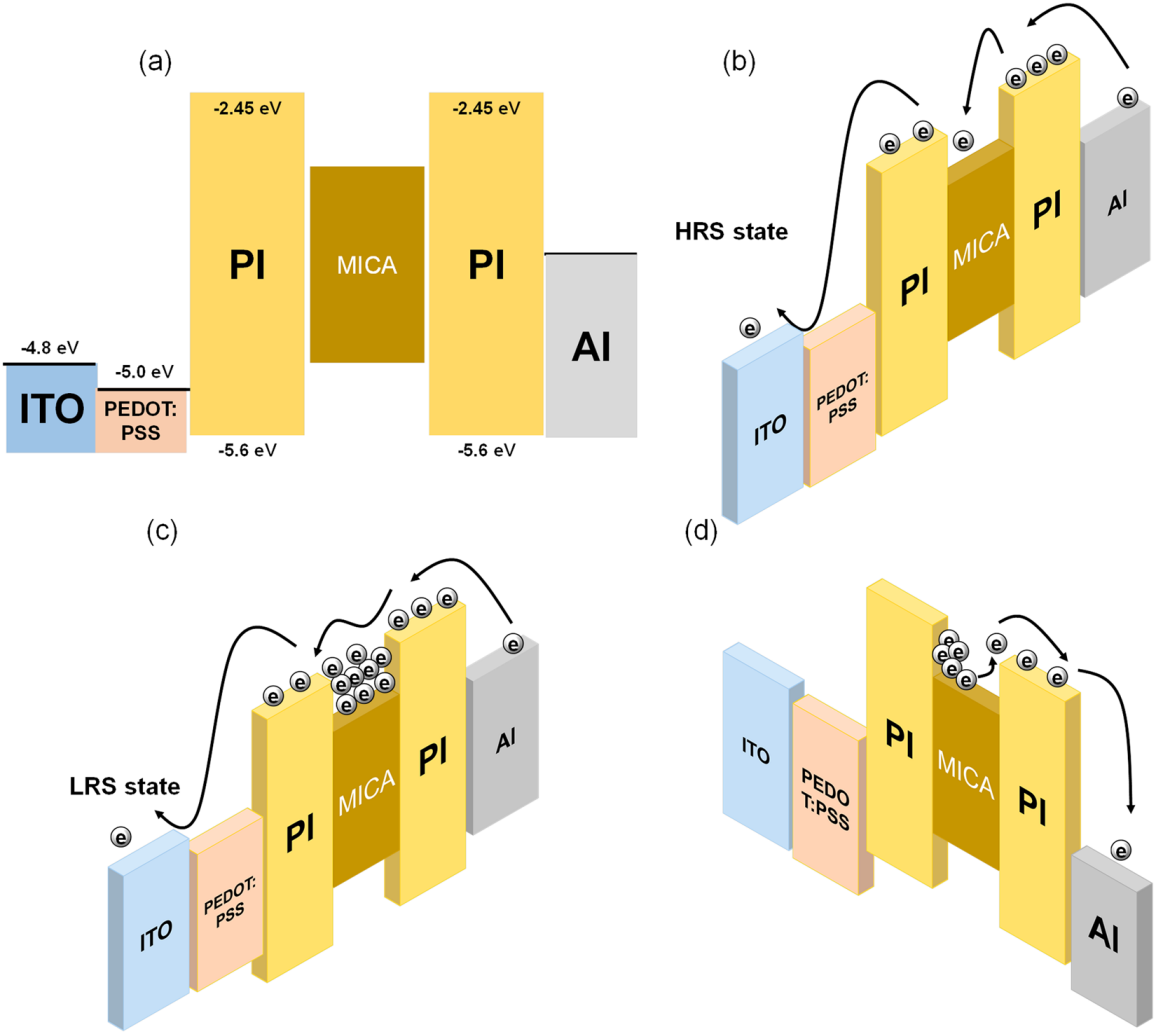
(**a**) Schematic energy band diagrams of the Al/PI:mica/PEDOT:PSS/ITO/PEN device. Schematic diagrams of the charge movement under (**b**) low and (**c**) high negative bias voltages in the HRS and (**d**) positive bias voltages in the LRS.

The set/reset operation mechanisms of the memristive device based on the charge-trapping mechanism are illustrated in Fig. 6(a–d). One should note that the mica nanosheets employed in this work had layer numbers ranging from 1 to 3 and bandgap energies ranging from 2.5 to 3.2 eV^14^. The trapping procedure corresponds to the memory process. The mica nanosheets act as electron trapping sites because the energy level of the conduction band for the mica nanosheets is lower than the lowest unoccupied orbital (LUMO) level of the PI layer, as shown in Fig. 6(a). The electrons emitted from the Al electrode in the HRS are partly captured in the mica nanosheets as trapped electrons, so the HRS current of the device at low voltages can be attributed to the SCLC process, as shown in Fig. 6(b)^30–32^. When higher voltages are applied, the number of injected electrons increases, and more electrons are captured by the mica nanosheets. As a result, the electron occupation probability for the mica increases as the Fermi level shifts to the LUMO level of the PI layer^30–32^. When the trap sites are fully occupied by electrons, the device switches from the HRS to the LRS, as shown in Fig. 6(c)^33^. The LRS current is attributed to the Ohmic process because of the trap-filled state and the possible presence of filamentary channels^34^. Even after the applied voltage is removed, the device remains in the LRS, indicative of non-volatility. When a positive voltage is applied, the device remains in the LRS until the reset voltage approaches 3 V. When that happens, the electrons trapped in the mica are emitted due to the positive voltage, as shown in Fig. 6(d), which corresponds to the erase process in the memory^33^.

## Conclusion

Flexible memristive devices based on PI:mica nanocomposites were investigated due to their superior mechanical flexibility. The bistable characteristics of the memristive devices were investigated in order to understand their stabilities and conduction mechanisms. The mica nanosheets embedded in the PI layer enhanced the resistive switching characteristics. The LRS/HRS current ratio for the devices before bending was 4.28 × 10^3^, and the LRS/HRS current ratios after bending at radii of 20 and 10 mm were 7.67 × 10^2^ and 1.08 × 10^2^, respectively. The endurance and the retention results before and after bending demonstrated that the flexible devices were stable and reliable. According to the fittings of the I–V curves, the carrier transport mechanism in the LRS could be attributed to Ohmic conduction and those in the HRS to Ohmic and SCLC behaviors. Furthermore, the mechanisms of the devices could be explained by using an energy band diagram. These results indicate that mica nanosheets should be useful for potential applications in flexible memristive devices.
